# Naringin attenuates fructose-induced NAFLD progression in rats through reducing endogenous triglyceride synthesis and activating the Nrf2/HO-1 pathway

**DOI:** 10.3389/fphar.2022.1049818

**Published:** 2022-12-15

**Authors:** Sirinat Pengnet, Phinsuda Sumarithum, Nuttaphong Phongnu, Sakdina Prommaouan, Napapas Kantip, Ittipon Phoungpetchara, Wachirawadee Malakul

**Affiliations:** ^1^ Division of Physiology, School of Medical Sciences, University of Phayao, Phayao, Thailand; ^2^ Department of Physiology, Faculty of Medical Science, Naresuan University, Phitsanulok, Thailand; ^3^ Department of Anatomy, Faculty of Medical Science, Naresuan University, Phitsanulok, Thailand; ^4^ Centre of Excellence in Medical Biotechnology, Naresuan University, Phitsanulok, Thailand

**Keywords:** naringin, fructose, non-alcoholic fatty liver disease, inflammation, *de novo* lipogenesis

## Abstract

**Background:** Excessive fructose consumption causes hepatic lipid accumulation *via* increased triglyceride (TG) synthesis, leading to the development and progression of non-alcoholic fatty liver disease (NALFD). Naringin, a flavanone glycoside found in citrus fruit, has antioxidant and hypolipidemic properties. Therefore, the aim of this study was to investigate the effect of naringin on fructose-induced NAFLD in rats and the possible underlying mechanism.

**Methods:** Male Sprague Dawley rats were given 10% (w/v) fructose in drinking water for 12 weeks. Naringin (100 mg/kg/day) was administered orally to rats for the last 4 weeks of fructose overload. After 12 weeks of treatment, the hepatic lipid content was determined. In addition, the expression of proteins involved in *de novo* lipogenesis (DNL) and TG synthesis as well as antioxidant and inflammatory mediators in the liver were examined by western blot analysis.

**Results:** Treatment of fructose-fed rats with naringin significantly decreased the hepatic TG and cholesterol content as well as serum aspartate aminotransferase (AST) and alanine aminotransferase (ALT) activities. Naringin treatment also decreased the hepatic expression of carbohydrate response element binding protein (ChREBP), sterol regulatory element-binding protein-1c (SREBP-1c) and nuclear SREBP-1c (nSREBP-1c) as well as enzymes involved in DNL (acetyl CoA carboxylase [ACC] and fatty acid synthase [FAS]) and an enzyme involved in TG synthesis (glycerol-3-phosphate acyltransferase 1 [GPAT-1] and diacylglycerol acyltransferase2 [DGAT2]) in fructose-fed rats. In addition, naringin induced a significant decrease in the hepatic expression of nuclear factor kappa B (NF-κB) and tumor necrosis factor α (TNF-α). Furthermore, naringin administration restored the expression of the antioxidant mediators nuclear factor (erythroid-derived 2)-like 2 (Nrf2) and heme oxygenase-1 (HO-1) in the liver of fructose-fed rats.

**Conclusion:** These results demonstrate that oral administration of naringin protects against fructose-induced hepatic steatosis by decreasing DNL and TG synthesis. In addition, naringin could prevent NAFLD progression *via* targeting the Nrf2/HO-1 and the NF-κB/TNF-α pathways.

## 1 Introduction

Non-alcoholic fatty liver disease (NAFLD), one of the most common chronic liver diseases, is characterized by excessive triglyceride (TG) accumulation as lipid droplets in the cytoplasm of hepatocytes in the absence of excessive alcohol intake (Rada et al., 2020). NAFLD is a major risk factor for the development of cirrhosis and possible subsequent hepatocellular carcinoma (Bertot and Adams, 2019). NAFLD begins with excessive TG in the liver (steatosis), which can progress to non-alcoholic steatohepatitis (NASH) (Pierantonelli and Svegliati-Baroni, 2019). This condition is characterized by fat deposits, inflammation and damage to hepatocytes. The prevalence of NAFLD has increased rapidly worldwide because of changes in lifestyle and dietary habits (Yasutake et al., 2014). Obesity and the intake of foods high in sugar and fat are closely associated with the development and progression of NAFLD (Eng and Estall, 2021). Recent studies in humans and animals have shown that high dietary intake of fructose is an important cause of hepatic steatosis (Abdelmalek et al., 2010; Alwahsh et al., 2014; Bray and Popkin, 2014; Sellmann et al., 2015). High fructose intake causes excessive lipid accumulation in the liver *via* numerous pathways, among which increased lipid synthesis plays a pivotal role (Jegatheesan and De Bandt, 2017). Activation of hepatic *de novo* lipogenesis (DNL) can contribute to the production and accumulation of TG in the liver, and this activation is regulated by several transcription factors (Jegatheesan and De Bandt, 2017). The two major mediators of pathways involved in hepatic DNL are carbohydrate response element-binding protein (ChREBP), which is a glucose-sensing transcription factor, and sterol regulatory element binding protein-1c (SREBP-1c), which is an insulin-sensing transcription factor (Jegatheesan and De Bandt, 2017). These two mediators operate mainly by upregulating lipogenesis-related genes, and this upregulation has been observed in the liver of rats with NAFLD (Jegatheesan et al., 2015). The glycerol-3-phosphate (G3P) pathway is another important pathway for triglyceride (TGs) synthesis in the liver, and is under the control of two key enzymes, i.e. acyl-CoA:glycerol-sn-3-phosphate acyltransferase (GPAT-1) and diacylglycerol/acyl-CoA acyltransferase-2 (DGAT2) (Gluchowski et al., 2019; Karasawa et al., 2019). GPAT-1 catalyzes the first step in TG synthesis, and DGAT2 is a rate-limiting enzyme catalyzing the final step in TG synthesis. Previous studies have demonstrated that the suppression of GPAT-1 and DGAT-2 reverses diet-induced hepatic steatosis in experimental animals (Park et al., 2011; Zhao et al., 2015; Gluchowski et al., 2019). Increased hepatic lipid accumulation promotes inflammatory cytokine production and oxidative stress, which contribute to the progression of hepatic steatosis to NASH and fibrosis (Jegatheesan and De Bandt, 2017; Eng and Estall, 2021). Therefore, improving lipid metabolism and/or reducing hepatic oxidative stress and inflammation are considered an effective strategy to prevent NAFLD progression. Nuclear factor (erythroid-derived 2)-like 2 (Nrf2) is a cytoprotective transcription factor that plays a major role in the elimination of ROS and subsequent reduction in the inflammatory response (Tu et al., 2019; Galicia-Moreno et al., 2020). It activates elements of the antioxidant response (ARE) and upregulates the expression of several antioxidant genes, including heme oxygenase-1 (HO-1) (Galicia-Moreno et al., 2020). The induction of HO-1, one subtype of the heme oxygenases, catalyzes the rate limiting step in the breakdown of heme to the antioxidant bilirubin (Origassa and Câmara, 2013). HO-1 and its metabolites have been shown to exert several protective biological activities, displaying antioxidant, anti-inflammatory and anti-apoptotic properties (Guo et al., 2018; Kuo et al., 2020). Recently, it has been demonstrated that an increased Nrf2/HO-1 signaling might play a significant role in preventing hepatic oxidative stress and inflammation induced by a high-fructose diet (Hu et al., 2017; Zhao et al., 2018).

Naringin (4′,5,7-trihydroxyflavone 7-rhamnoglucoside) is found in grapefruit and related citrus species; its colonic metabolite is naringenin (Arafah et al., 2020). Both naringin and naringenin have been reported to display a number of pharmacological activities, including antioxidant, anti-inflammatory, hypoglycemic, hypolipidemic, cardioprotective and hepatoprotective effects (Ortiz-Andrade et al., 2008; Jain and Parmar, 2011; Alam et al., 2013; Hermenean et al., 2014; Ahmed et al., 2017; Rashmi et al., 2018; Chung et al., 2019; Pengnet et al., 2019; Yu et al., 2019). Previous studies have reported that naringin can ameliorate high-fat-diet-induced hepatic steatosis by decreasing oxidative stress, promoting fatty acid oxidation, inhibiting fatty acid synthesis in the liver (Pu et al., 2012; Mu et al., 2020), and modulating intestinal gut microbiota (Mu et al., 2020). In addition, our previous study indicated that daily administration of naringin at 100 mg/kg/day for 4 weeks can lower serum levels of TG, TC, and LDL-C in high fructose solution-fed rats. This finding also demonstrated the vascular-protective properties of naringin *via* decreasing vascular oxidative stress (Malakul et al., 2018). Growing evidence suggests that the lipid-lowering properties of natural products may be associated with the amelioration of NAFLD (Malakul et al., 2022; Prommaouan et al., 2022). However, the efficacy of naringin in preventing NAFLD progression in fructose-induced NAFLD has not yet been evaluated. Therefore, the purpose of the current study was to determine whether naringin could ameliorate NAFLD progression induced by fructose feeding, and to evaluate the hypothesis that naringin inhibits the progression of NAFLD *via* regulating the DNL and G3P pathways, enhancing the Nrf2/HO-1 pathway and decreasing inflammatory cytokine production.

## 2 Materials and methods

### 2.1 Animal experiments

All animal procedures for the study were approved by the Institutional Animal Care Committee of Naresuan University (ethical approval No. NUAE57040023). Male Sprague Dawley rats (weighing 180–200 g) were procured from the National Laboratory Animal Center at Salaya, Mahidol University, Thailand. All rats were maintained in the Center for Animal Research at Naresuan University at a controlled ambient temperature (22 ± 1°C), with a 12-hour photoperiod. After a week of acclimatization, the rats were divided randomly into four groups as follows (n = 8 rats/group): Group I, control group (C); these rats received normal drinking water. Group II, fructose group (HF); these rats received fructose solution and the vehicle 0.1% carboxymethylcellulose (CMC). Group III, fructose plus naringin group (HFN); these rats received fructose solution and naringin suspended in 0.1% CMC. Group IV, control plus naringin (CN); these rats received normal drinking water and naringin. The fructose solution was prepared as described previously (Malakul et al., 2018) and given to the rats every day for 12 weeks. Naringin (100 mg/kg/day) or the vehicle (0.1% CMC) was administered daily by oral gavage during the last 4 weeks of fructose feeding. This dosage was chosen based on previous results obtained by our group, which indicated the lipid-lowering activity of naringin in fructose-fed rats (Malakul et al., 2018).

After 12 weeks, all rats were anesthetized with an intraperitoneal injection of sodium pentobarbital (50 mg/kg) and a blood sample were collected *via* cardiac puncture. The serum was separated by centrifugation and was stored at −80°C for biochemical assays. The abdominal cavity was opened and the liver was removed, weight, and then washed in cold phosphate-buffered saline (PBS) to remove surface blood. Tissues were blotted to remove buffer, snap-frozen in liquid nitrogen and stored at −80°C for subsequent experiments.

### 2.2 Determination of the hepatic lipid contents and hepatic function markers

The total lipid content was extracted from fresh frozen liver tissues by using a lipid extraction kit (CAS Number: ab211044; Abcam, Cambridge, United Kingdom) according to the manufacturer’s protocols. The TG and TC contents in the liver were then determined enzymatically using commercial test kits (Sigma-Aldrich; Merck KGaA, Darmstadt, Germany) according to the manufacturer’s instructions. The total TG and TC levels are expressed as mg/g liver.

The activities of aspartate aminotransferase (AST) and alanine aminotransferase (ALT) are commonly used to assess liver function and injuries. Serum levels of AST and ALT were analyzed quantitatively by using a GOT (ASAT) or GPT (ALAT) activity assay kit (HUMAN Diagnostics Worldwide, Wiesbaden, Germany) according to the manufacturer’s protocols.

### 2.3 Determination of hepatic lipid accumulation

As previously described (Malakul et al., 2022), the liver tissues were embedded in optimal cutting temperature compound and were cryosectioned at 8 μm. The sections were stained in Oil Red O solution and then counterstained with hematoxylin. The percentage of Oil Red O stained area were measured using the ImageJ analyzing system (version 1.51j, National Institutes of Health, United States).

### 2.4 Determination of ROS levels in the liver

Rat livers were frozen in optimum cutting temperature compound, cryosectioned, and stained with dihydroethidium (DHE) for 30 min at 37°C in the dark. After staining, sections were washed with PBS, and mounted in 50% glycerol. ROS levels in the liver were observed and indicated by a red fluorescent signal under a fluorescence microscope (Pengnet et al., 2019).

### 2.5 Determination of the expression of proteins related to lipogenesis, the antioxidant pathway and inflammation using western blot analysis

The expression of proteins involved in lipogenesis (ChREBP, SREBP-1c, nSREBP-1c, fatty acid synthase [FAS], acetyl CoA carboxylase [ACC], GPAT-1 and DGAT2), the antioxidant pathway (Nrf2 and HO-1) and inflammation (nuclear factor kappa B [NF-κB] and tumor necrosis factor alpha [TNF-α]) in the liver were assessed. Liver samples (100 mg each) were homogenized in cold radioimmunoprecipitation assay (RIPA) lysis buffer with a 1% protease inhibitor cocktail, incubated for 30 min and then centrifuged at 20,000 g for 30 min at 4°C. The whole cell lysates were fractionated into nuclear components, as previously described by Ontawong et al. (Ontawong et al., 2013). Briefly, liver tissues were homogenized in RIPA lysis buffer containing a 1% protease inhibitor cocktail and then centrifuged at 5,000 g for 10 min at 4°C. Afterward, the pellet was resuspended in the same solution, centrifuged at 10,000 g for 10 min at 4°C, and designated as a nuclei-enriched fraction. The protein concentration of the supernatants was determined by using a bicinchoninic acid (BCA) assay kit (Merck KGaA, Darmstadt, Germany) and measuring the absorbance at 562 nm. Protein samples (80 μg) were loaded onto 12.5% (w/v) gels and subjected to sodium dodecyl sulphate-polyacrylamide gel electrophoresis (SDS-PAGE) for 1 h. The separated protein was transferred onto polyvinylidene fluoride (PVDF) membranes (Millipore, Bedford, MA) using a Trans-Blot^®^ SD Semi-Dry Transfer Cell (Bio-Rad laboratories, Hercules, CA, United States), and the membranes were incubated in 5% non-fat dry milk in Tris-buffered saline plus 0.1% Tween 20 (TBS-T) for 1 h. After washing in TBS-T, the membranes were subsequently incubated with the specific primary antibodies in TBS-T buffer containing 5% bovine serum albumin overnight at 4°C. The primary antibodies used were: anti-GPAT-1 diluted 1:1000, anti-DGAT2 diluted 1:1000, anti- ChREBP diluted 1:1000, anti-SREBP-1c diluted 1:1000, anti-FAS diluted 1:2500, anti-ACC diluted 1:2500, anti-Nrf2 diluted 1:2000, anti-HO-1 diluted 1:1000, anti-NF-κB diluted 1:1000, anti-TNF-α diluted 1:1000, anti-β-actin diluted 1:5000 and anti-Lamin B1 diluted 1:2000. The membrane was washed with TBS-T and the incubated with horseradish-peroxidase-conjugated secondary goat anti-rabbit or anti-mouse IgG (Merck KGaA, Darmstadt, Germany) in TBS-T buffer containing 5% non-fat dried milk for 1 h. The membranes were then incubated in an enhanced chemiluminescence solution (Bio-Rad Laboratories Inc., United States) and the protein bands were analyzed quantitatively by using the Image Lab™ software (Bio-Rad Laboratories Inc., United States).

### 2.6 Drugs and chemicals

Naringin (purity >90%), DHE (CAS Number: 104821-25-2) and the primary antibody against DGAT2 (Product number: SAB2106887, Lot number: QC29804) were purchased from Sigma-Aldrich (St. Louis, MO, United States). Primary antibodies against ACC (CAS Number: 05-1098), Nrf2 (CAS Number: MABE1799), HO-1 (CAS Number: AB1284), NF-κB (CAS Number: ABE347), TNF-α (CAS Number: AB 1837P) and Lamin B1 (CAS Number: MABS492) were purchased from Merck KGaA (Darmstadt, Germany). The primary antibody against ChREBP (Product number: OAAN03715) was purchased from Aviva System Biology (San Diego, CA, United States). The primary antibodies against SREBP-1c (CAS Number: PA1-46142), FAS (CAS Number: MA5-14887) and β-actin (CAS Number: MA5-15739) were purchased from Thermo Fisher Scientific (Waltham, MA, United States). The primary antibodies against GPAT-1 (CAS Number: ab69990) was purchased from Abcam (Waltham, MA, United States).

### 2.7 Statistical analyses

Data are expressed as the mean ± standard error of the mean. The data were analyzed using one-way analysis of variance followed by the Tukey’s multiple comparison test with GraphPad Prism version 5 (GraphPad Software, San Diego, CA, United States). Statistical significance was accepted for *p* < 0.05.

## 3 Results

### 3.1 Naringin treatment reduced fructose-induced hepatic steatosis

**FIGURE 1:**
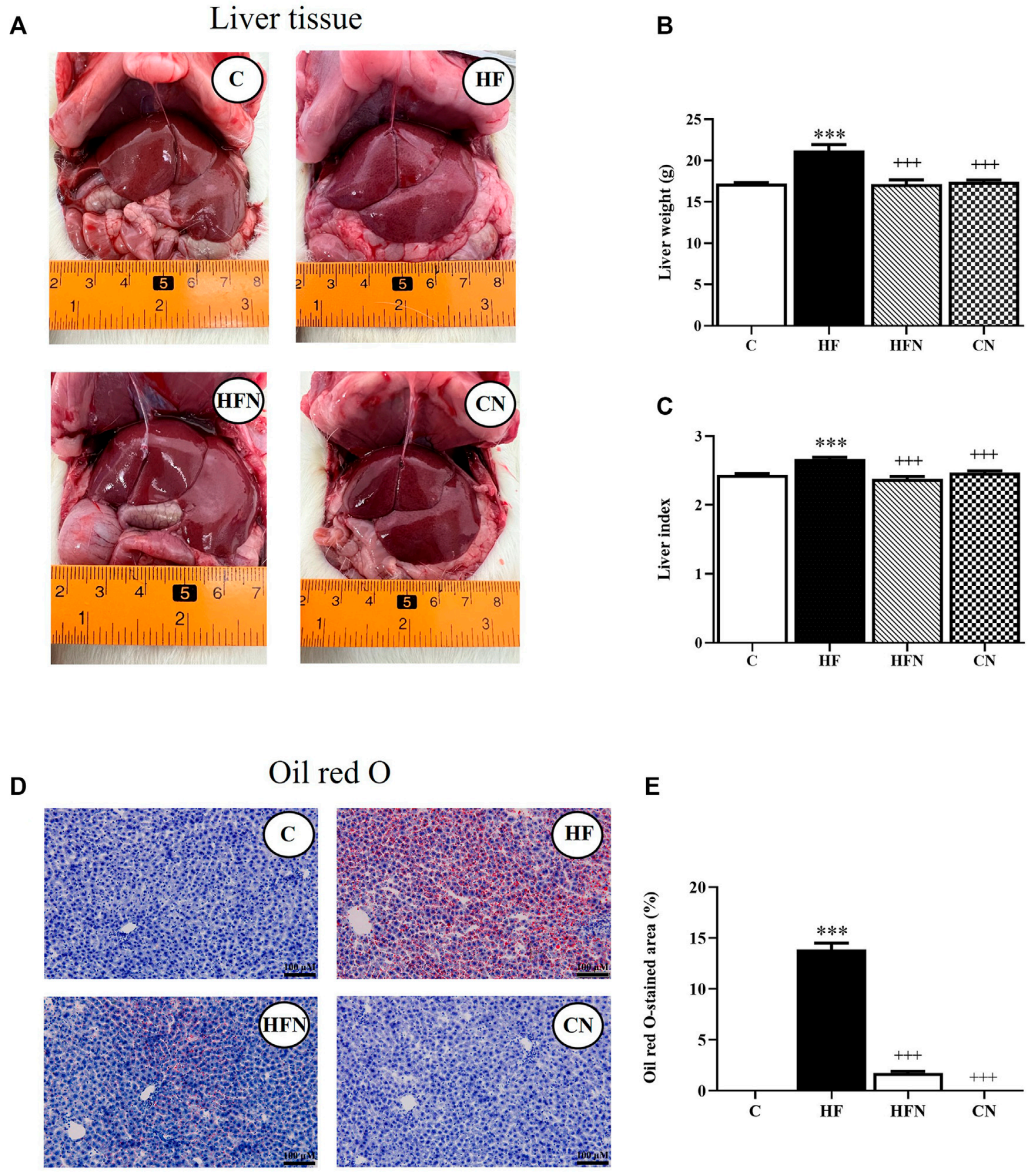
The effects of naringin on hepatic lipid accumulation. **(A)** Macroscopic appearance, **(B)** liver weight, **(C)** liver index, **(D)** Oil Red O staining of livers (magnification ×20) and **(E)** the percentage of Oil Red O-stained liver section in control rats (C), fructose-fed rats (HF), fructose-fed rats treated with naringin at 100 mg/kg (HFN) and control rats treated with naringin at 100 mg/kg (CN). Liver index was calculated using the formula [(liver weight/body weight)*100]. The Oil Red O staining shows as red-orange, defining the lipid deposition area, and hematoxylin staining in blue indicated the cell nucleus. The results shown are presented as the mean ± standard error of the mean (n = 5 per group). ^***^ *p* < 0.001 compared with the control group; ^+++^ *p* < 0.001 compared with the HF group.

To evaluate the effects of naringin on hepatic lipid accumulation, we examined liver morphology in fructose-induced hepatic steatosis after treatment with naringin for 4 weeks. The results showed that the rats that received 10% fructose in their drinking water for 12 weeks (the HF group) showed a significant increase in the liver weight and index compared with the C group (*p* < 0.001) (Figures 1B,C). Four weeks of supplementation with naringin at 100 mg/kg/BW (the HFN group) significantly decreased liver weight and index compared with the HF group (*p* < 0.001) but did not affect these levels in the C group. Oil Red O staining showed that treatment of fructose-fed rats with naringin significantly decreased the percentage of the Oil Red O-stained area in the liver sections (*p* < 0.001) (Figures 1D,E); however, this treatment did not alter these values in control rats.

**FIGURE 2:**
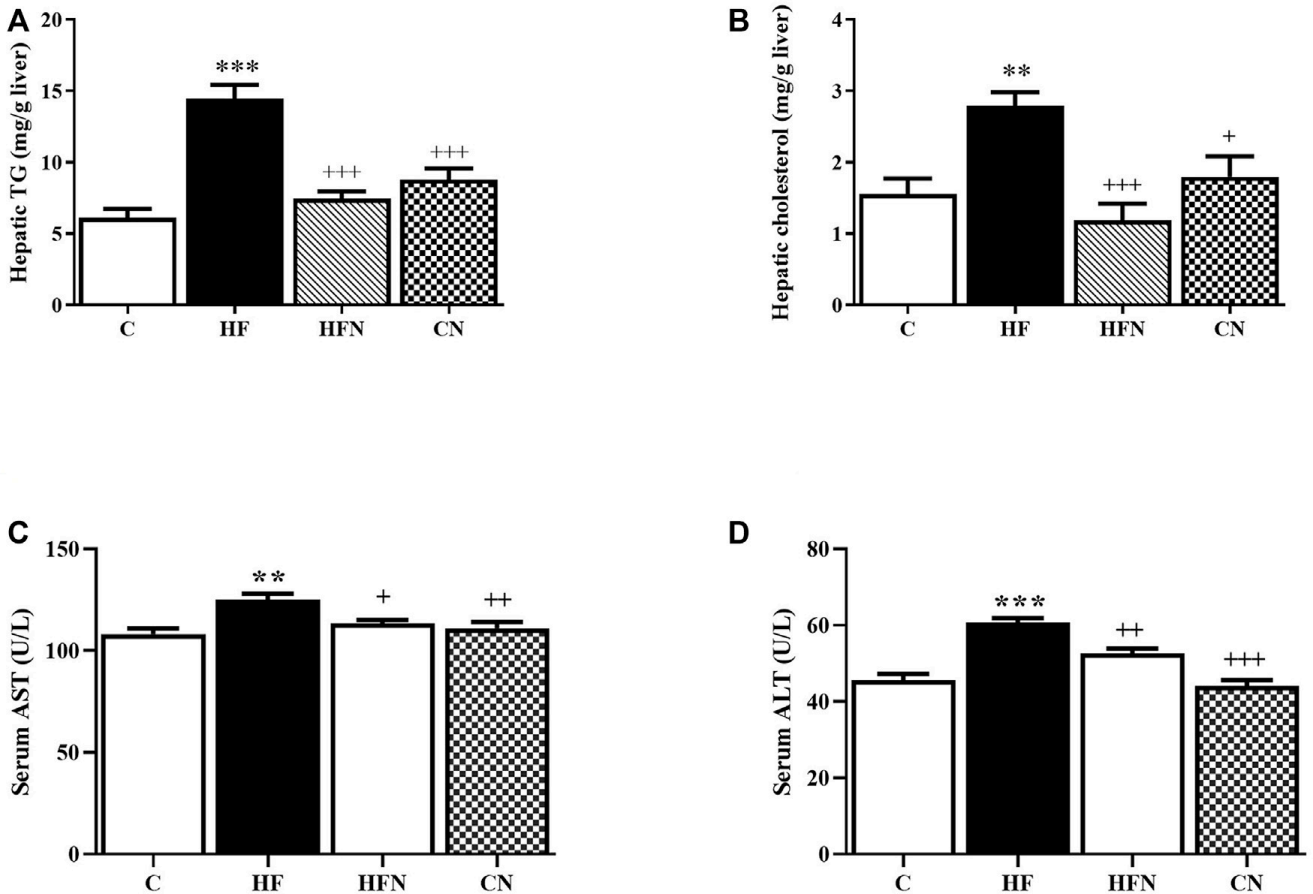
The effects of naringin on hepatic lipid content and liver function. **(A)** Hepatic total triglycerides level, **(B)** hepatic total cholesterol level, **(C)** Aspartate amino-transferase (AST) and **(D)** alanine amino-transferase (ALT) activities in serum of control rats (C), fructose-fed rats (HF), fructose-fed rats treated with naringin at 100 mg/kg (HFN) and control rats treated with naringin at 100 mg/kg (CN). The results shown are presented as the mean ± standard error of the mean (n = 5 per group). ^**^ *p* < 0.01, ^***^ *p* < 0.001 compared with the control group; ^+^ *p* < 0.05, ^++^ *p* < 0.01, ^+++^ *p* < 0.001 compared with the HF group.

To further confirm the ant-steatotic effects of naringin, hepatic lipid contents were determined. The HF group showed a significant increase in hepatic TG and TC levels compared with the C group (*p* < 0.001, 0.01) (Figures 2A,B). The treatment with naringin significantly reduced the hepatic TG and TC levels compared with the HF group (*p* < 0.001). These results indicate that naringin reduces high fructose-induced hepatic steatosis.

### 3.2 Naringin treatment ameliorated fructose-induced hepatic injuries

As shown in Figures 2C,D, the serum levels of AST and ALT, hepatic damage markers, of the HF group were significantly increased compared with the C group (*p* < 0.01, 0.001). Naringin treatment did not affect both AST and ALT activities in control rats, but significantly decreased these values of fructose-fed rats (*p* < 0.05, 0.01). These findings suggest a beneficial effect of naringin on high fructose-induced liver injury.

### 3.3 Naringin treatment inhibited ChREBP/SREBP1c-mediated DNL

**FIGURE 3:**
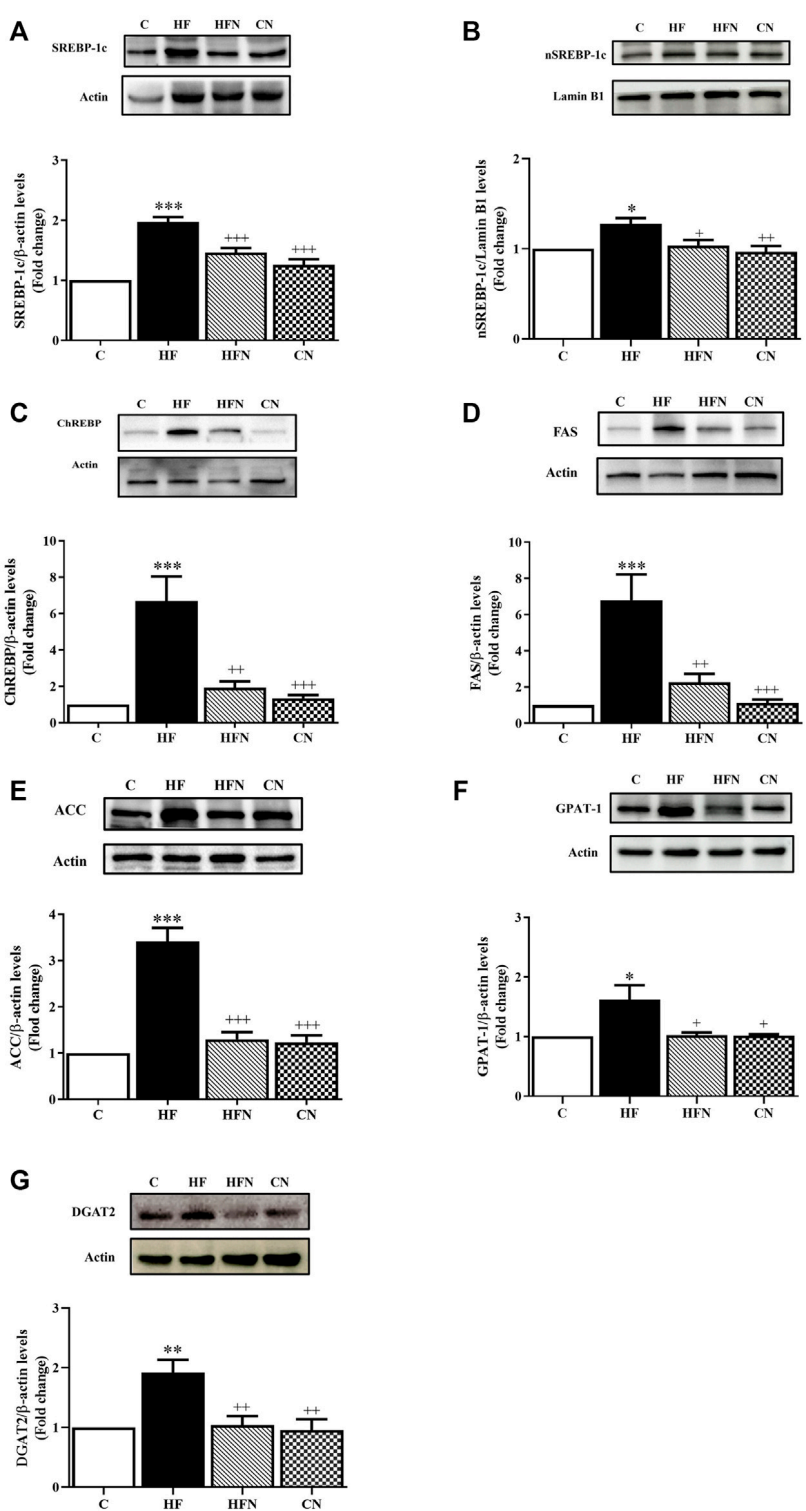
The effects of naringin on hepatic lipogenesis. Hepatic expression of **(A)** sterol regulatory element binding protein-1c (SREBP-1c), **(B)** nuclear SREBP-1c (nSREBP-1c), **(C)** carbohydrate response element-binding protein (ChREBP), **(D)** fatty acid synthase (FAS), **(E)** acetyl CoA carboxylase (ACC), **(F)** glycerol-3-phosphate acyltransferase 1 (GPAT-1) and **(G)** diacylglycerol/acyl-CoA acyltransferase-2 (DGAT2) in control rats (C), fructose-fed rats (HF), fructose-fed rats treated with naringin at 100 mg/kg (HFN) and control rats treated with naringin at 100 mg/kg (CN). The results are presented as the mean ± standard error of the mean (n = 5 per group). ^*^ *p* < 0.05, ^**^ *p* < 0.01, ^***^ *p* < 0.001 compared with the control group; ^+^ *p* < 0.05, ^++^ *p* < 0.01, ^+++^ *p* < 0.001 compared with the HF group.

To clarify the mechanism by which naringin reduced hepatic lipid accumulation in fructose-fed rats, we examined whether naringin affected the expression of SREBP-1c, nSREBP-1c and ChREBP as well as their downstream target proteins FAS and ACC in the liver. As shown in Figures 3A–E, the expression of SREBP-1c, nSREBP-1c, ChREBP, FAS and ACC was increased significantly in the HF compared with the C group. However, naringin treatment decreased the expression of all assessed proteins.

### 3.4 Naringin treatment alleviated the expression of the enzymes of triglyceride synthesis

To determine whether naringin treatment could affect triglyceride regulatory mechanisms in the liver, protein expression of triglyceride-related enzymes was evaluated by Western blotting. The results showed that the HF group showed a significant increase in the protein expression of GPAT-1 and DGAT2 compared with the C group (*p* < 0.05, 0.01). However, treatment of fructose-fed rats with naringin decreased those protein (Figures 3F,G). In addition, there were no significant differences in the expression of GPAT-1 and DGAT2 between the C and CN groups.

### 3.5 Naringin treatment down-regulated the inflammatory response induced by high fructose

**FIGURE 4:**
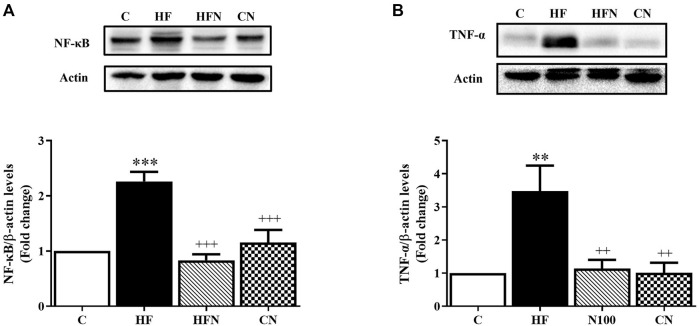
The effects of naringin on anti-inflammatory mediator expression. Hepatic protein expression of **(A)** nuclear factor kappa B (NF-κB) and **(B)** tumor necrosis factor α (TNF-α) in control rats (C), fructose-fed rats (HF), fructose-fed rats treated with naringin at 100 mg/kg (HFN) and control rats treated with naringin at 100 mg/kg (CN). The results are presented as the mean ± standard error of the mean (n = 5 per group). ^**^ *p* < 0.01, ^***^ *p* < 0.001 compared with the control group; ^++^ *p* < 0.01, ^+++^ *p* < 0.001 compared with the HF group.

To further determine the effect of naringin on fructose-induced NAFLD progression in rats, the protein expression of NF-κB and TNF-α was determined in the liver. The expression of NF-κB and TNF-α was increased significantly in the liver of the HF compared with the C group (*p* < 0.001, 0.01) (Figures 4A,B). After naringin administration for 4 weeks, the hepatic expression of NF-κB and TNF-α had decreased significantly in the HFN group compared with the HF group (*p* < 0.001, 0.01). However, there were no significant differences between the C and CN groups in the hepatic expression of NF-κB and TNF-α. These results demonstrate that naringin treatment improves fructose-induced hepatic inflammation.

### 3.6 Naringin treatment accelerated the expression of Nrf2 and HO-1, and reduced ROS generation

**FIGURE 5:**
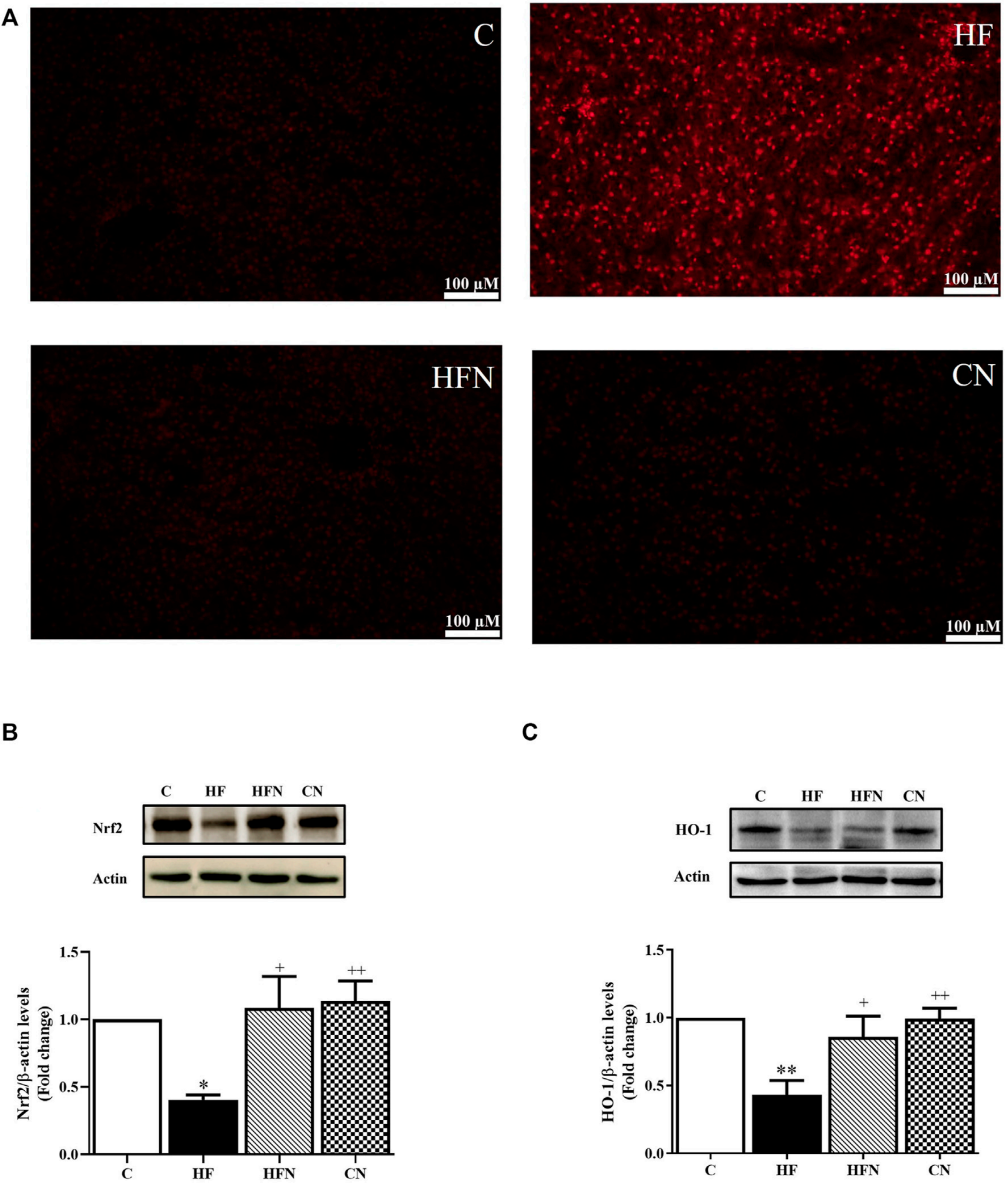
The effects of naringin on hepatic oxidative stress. **(A)** A representative the oxidative dye DHE staining images of liver section (magnification ×20), hepatic protein expression of **(B)** nuclear factor (erythroid-derived 2)-like 2 (Nrf2) and **(C)** heme oxygenase-1 (HO-1) of control rats (C), fructose-fed rats (HF), fructose-fed rats treated with naringin at 100 mg/kg BW (HFN) and control rats treated with naringin at 100 mg/kg BW (CN). The results are shown as the mean ± standard error of the mean (n = 5 per group). ^*^ *p* < 0.05, ^**^ *p* < 0.01 compared with the control group; ^+^ *p* < 0.05, ^++^ *p* < 0.01 compared with the HF group.

To evaluate the possible role of naringin in hepatic oxidative stress, ROS generation and the protein expression of Nrf2 and HO-1 was determined in the liver. Figure 5A shows that DHE fluorescence intensity, an indicator of ROS, was higher in liver sections from HF group than in the control group, whereas naringin treatment attenuated the DHE fluorescence intensity to control levels. Western blot analysis revealed that fructose-fed rats showed a significant decrease in Nrf2 and HO-1 protein expression in the liver (*p* < 0.05, 0.01), whereas these proteins were significantly increased after naringin treatment (*p* < 0.05) (Figures 5B,C). However, there were no significant differences in DHE staining and the expression of Nrf2 and HO-1 between the C and CN groups. These results demonstrate that naringin reduces oxidative stress caused by high fructose consumption.

## 4 Discussion

The present results clearly demonstrate that treatment of rats with naringin, a major bioflavonoid in citrus fruits, was highly effective in preventing fructose-induced fatty liver disease. This could be due to its effect on decreasing the protein levels of ChREBP, SREBP-1c, nSREBP-1c and their target proteins, ACC and FAS, with a prominent decrease in GPAT-1 and DGAT2 expression. In addition, our study is the first to demonstrate that naringin can prevent the progression of NAFLD by suppressing the NF-kB/TNF-α pathway and increasing the Nrf2/HO-1 pathway in the liver of fructose-induced NAFLD rats.

Fructose is commonly used in large amounts throughout the world as a sweetener in food and beverages. After fructose consumption, it is metabolized rapidly in the liver and provides large amounts of hepatic triose-phosphate as a substrate for hepatic DNL (Federico et al., 2021). This process converts carbohydrates into fatty acids by the key enzymes ACC and FAS; these fatty acids are esterified into TG, which are then exported from the liver as very-low-density lipoprotein (VLDL) (Alves-Bezerra and Cohen, 2017). TG may accumulate as lipid droplets in the cytoplasm of hepatocytes due to increased DNL; the consequence of this phenomenon is fatty liver (Alves-Bezerra and Cohen, 2017; Gerges et al., 2021). Several studies suggest that overconsumption of dietary fructose causes lipid deposition in the liver and leads to the development of NAFLD (Alwahsh et al., 2014; Sellmann et al., 2015; Li et al., 2018; Zhao et al., 2018).

Natural compounds found in fruits and vegetables, such as flavonoids, have been shown to ameliorate hepatic lipid accumulation, thus providing excellent therapeutic potential for the treatment of NAFLD (Braud et al., 2017). Several studies have demonstrated that naringin possesses great health benefits, and prevents various metabolic diseases, such as hyperlipidemia, cardiovascular dysfunction and obesity (Pu et al., 2012; Alam et al., 2013; Pengnet et al., 2019). We previously reported that the beneficial effects of oral administration of naringin on serum lipid levels in fructose-fed rats (Malakul et al., 2018); therefore, we hypothesized that naringin could prevent fructose-induced NAFLD because of its anti-hyperlipidemic properties. Previous study has reported that naringin is able to ameliorate NAFLD induced by high-fat diets in experimental animals by modulating the intestinal bacterial composition (Mu et al., 2020). However, the mechanisms by which naringin acts on hepatic lipid accumulation has not been elucidated clearly. In the present study, we further investigated the influences of naringin administration on lipid accumulation in the liver and related mechanisms in fructose-fed rats. Consistent with other findings (Li et al., 2018; Yang et al., 2019), we found that a 10% solution of fructose in the drinking water promoted hepatic steatosis, as demonstrated by an increase in liver weight, the liver/body weight ratio, Oil Red O staining area of the liver and hepatic TG and TC contents. In addition, the activities of serum AST and ALT, as indicators of liver function and liver injury, were increased in the high-fructose group. These results suggest that high fructose intake induced liver steatosis and damage, resulting in the development NAFLD. In this study, naringin treatment effectively reversed the fructose-induced alterations in these parameters, indicating that naringin improved liver steatosis and damage induced by fructose feeding.

Accumulating evidence has demonstrated that the principal route for fructose feeding-induced hepatic steatosis is the increase in DNL, and a rate-limiting enzyme for this pathway is ACC and FAS (Silva et al., 2019; Federico et al., 2021). Both ACC and FAS are known to be regulated by the transcription factors SREBP-1c and ChREBP (Alves-Bezerra and Cohen, 2017). SREBP-1c is a lipogenic transcriptional factor that is translocated into the nucleus for stimulating the DNL in response to a high-fat or high-carbohydrate diet, and may act in synergy with ChREBP, a glucose-mediated transcription factor, strongly regulates the gene transcription in DNL in response to dietary carbohydrates (Li et al., 2011; Alves-Bezerra and Cohen, 2017; Lane et al., 2019; Ortega-Prieto and Postic, 2019). Consistent with other studies (Erion et al., 2013; Kim et al., 2019), we found increased ChREBP, SREBP-1c and nSREBP-1c expression as well as upregulated levels of their downstream targets, FAS and ACC, in the liver of fructose-fed rats. These results indicate that fructose consumption induced the synergistic action of ChREBP and SREBP-1c on hepatic DNL, which creates fatty acids that are assimilated into TG. In addition to the DNL pathway, the G3P pathway is a crucial source for TG synthesis, which contributes to over 90% of total TG synthesis; a rate-limiting enzyme for this pathway in the liver is GPAT-1 and DGAT2 (Gluchowski et al., 2019; Karasawa et al., 2019). GPAT-1 provides glycerol-3-phosphate as a substrate for diacylglycerol, which serves as a precursor molecule for TG synthesis (Karasawa et al., 2019). DGAT2 catalyzes the acylation of diacylglycerol, resulting in enhanced TG synthesis (Zhao et al., 2015).

Naringin has been reported to reduce lipid synthesis by reducing the hepatic expression of several proteins involved in the DNL pathway, such as SREBP-1, ACC and FAS in mice fed a high-fat diet (Pu et al., 2012), diabetic rats fed a high-fat diet (Kumar Sharma et al., 2011), alcoholic fatty liver disease in rats (Zhou et al., 2019) and in obese C57BL/6J mice (Mu et al., 2020). Our data showed that treatment with naringin reversed the upregulated hepatic expression of ChREBP, SREBP-1c, nSREBP-1c, FAS and ACC in fructose-fed rats. Another interesting observation in the current study was that naringin treatment in fructose-fed rats also reduced the protein levels of GPAT-1 and DGAT2. These results suggest that downregulating the expression of ChREBP, SREBP-1c, nSREBP-1c, FAS and ACC, as well as the downregulating GPAT-1 and DGAT2 expression, are critical mechanisms involved in the naringin-induced inhibition of TG synthesis in fructose-fed rats. This finding was consistent with our results illustrated in the liver morphology and hepatic lipid contents. Therefore, the reduced expression of DNL and G3P pathway-related proteins may contribute to the anti-steatotic effect of naringin. According to the two-hit hypothesis of NAFLD pathogenesis, oxidative stress and inflammation play important roles in the second-hit action, wherein simple steatosis can progress to NASH. During NAFLD, there is increased lipid accumulation in hepatocytes and oxidative stress is promoted. These alterations result in the release of local inflammatory cytokines that further exacerbate NAFLD (Rada et al., 2020). The nuclear transcription factor Nrf2, which is redox-sensitive, plays a crucial role in the modulation of proteins and enzymes against oxidative stress and inflammation, by activating the expression of several antioxidant enzymes (Park et al., 2011). During exposure to oxidative stress or inflammation, Nrf2 dissociates from its cytoplasmic inhibitor protein and subsequently translocates to the nucleus to activate the downstream transcription of various antioxidant enzymes, including HO-1 (Park et al., 2011). HO-1 is regarded as one of the most important intracellular antioxidant mechanisms; it converts heme to carbon monoxide (CO), ferrous iron and biliverdin (Duvigneau et al., 2019). Both CO and biliverdin exert potent cellular protective effects *via* their antioxidant and anti-inflammatory activities (Duvigneau et al., 2019). A number of studies have demonstrated that high fructose intake induces oxidative stress and inflammation in various tissues, including the liver, which may activate the Nrf2/HO-1 signaling pathway, a cellular antioxidant defense (Hu et al., 2017; Sharma et al., 2017; Zhao et al., 2018). Therefore, the pharmacological activation of Nrf2 expression has the potential to inhibit the progression of NAFLD. Consistent with previous findings (Hu et al., 2017; Sharma et al., 2017), we demonstrated increased ROS generation and down-regulated expression of Nrf2 and HO-1 in the liver of fructose-fed rats. The results indicate that hepatic oxidative stress can be induced in fructose-fed rat.

Previous studies demonstrated that naringin reduced oxidative stress through the activation of antioxidative pathways (Gopinath and Sudhandiran, 2012; Lv et al., 2018). Thus, we further investigated whether the Nrf2/HO-1 signaling pathway is involved in the mechanisms of the protective effect of naringin in high fructose-induced NAFLD. The results reveal that naringin administration in rats fed with a high-fructose solution up-regulated Nrf2 and HO-1 expression in the liver. As predicted, positive DHE staining, as a marker of ROS generation, was decreased in the liver section by naringin administration. This effect may have been attributable to the activation of the Nrf2/HO-1 signaling pathway.

Chronic oxidative stress, generated through the oxidation of cytotoxic free fatty acids, is closely linked to the inflammatory response in the liver. NF-κB is a critical transcription factor that regulates the production of inflammatory cytokines involved in the pathogenesis of inflammatory diseases and has been implicated in the development of the chronic inflammatory state in NAFLD (Chen et al., 2014). Experimental studies have shown that overconsumption of fructose activates NF-κB, which leads to the release of pro-inflammatory cytokines that develop into inflammatory responses (Bai et al., 2020; Heeba et al., 2021; Li et al., 2022). These findings are supported by our results, which revealed higher levels of NF-κB and pro-inflammatory cytokines (TNF-α) in the fructose-fed rats compared with the control rats. In this study, treatment with naringin reversed the upregulated hepatic expression of NF-κB and TNF-α in fructose-fed rats. Taken together, the findings of this study indicate that naringin can activate the Nrf2/HO-1 signaling pathway in the liver, and that these effects may contribute to the reduction of oxidative stress, inflammation and NAFLD progression.

## 5 Conclusion

**FIGURE 6:**
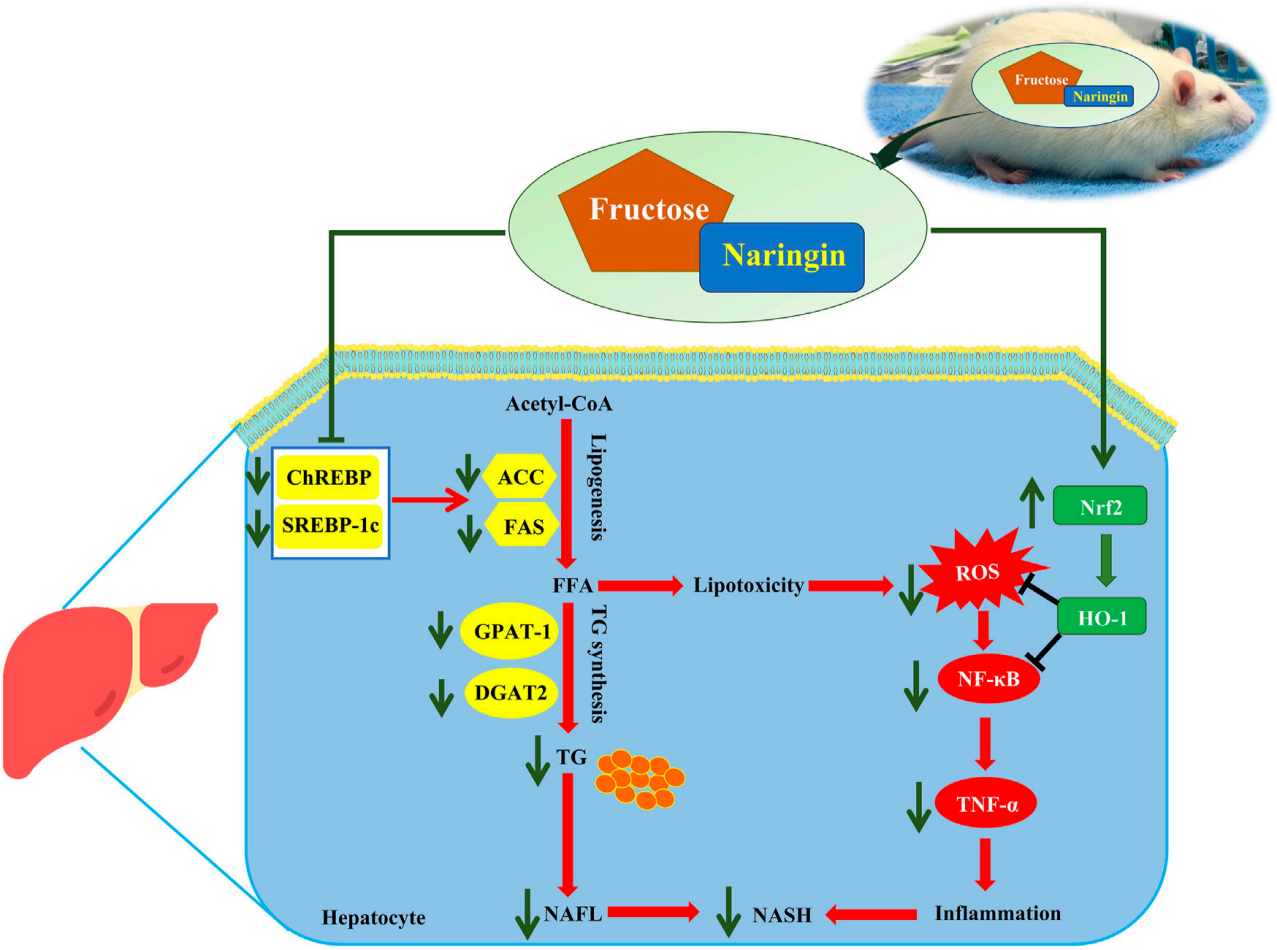
Schematic diagram of the possible mechanism of naringin in fructose-induced fatty liver disease.

The present findings demonstrate the effectiveness of treatment with naringin on hepatic steatosis in fructose-induced NAFLD in rats. Our study found that naringin reduced the hepatic DNL pathway by downregulating the protein levels of ChREBP, SREBP-1c, nSREBP-1c and their target proteins, ACC and FAS, and decreased the G3P pathway by downregulating the hepatic expression of GPAT-1 and DGAT2 in the liver. Thus, naringin protects against fructose-induced hepatic lipid accumulation. In addition, naringin protects against fructose-induced liver injury and inflammation in rats, in part by activating the Nrf2/HO-1 pathway and modulating the NF-κB pathway (Figure 6). Therefore, we have demonstrated that naringin has beneficial effects that prevent and improve fructose-induced NAFLD pathogenesis.

## Data Availability

The raw data supporting the conclusions of this article will be made available by the authors, without undue reservation.
